# THE SAFEST REVIEW: THE SHOCK-ABSORBING FLOORING EFFECTIVENESS SYSTEMATIC REVIEW IN CARE SETTINGS

**DOI:** 10.1093/geroni/igz038.1827

**Published:** 2019-11-08

**Authors:** Amy K Drahota, Bethany E Keenan, Chantelle Lachance, Lambert Felix, James P Raftery, Kirsten R Farrell-Savage, Dawn Mackey

**Affiliations:** 1 University of Portsmouth, Portsmouth, Hampshire, United Kingdom; 2 Cardiff University, Cardiff, Wales, United Kingdom; 3 St. Michael’s Hospital, Toronto, Ontario, Canada; 4 University of Southampton, Southampton, Hampshire, United Kingdom; 5 Simon Fraser University, Burnaby, British Columbia, Canada

## Abstract

Falls in hospitals and care homes are a major issue of international concern. Falls cost the US $34 billion a year, with injurious falls being particularly life-limiting and costly. Shock-absorbing flooring decreases the stiffness of the ground surface to reduce the impact of a fall. There is a growing body of evidence on flooring for fall-related injury prevention, however no systematic review exists to inform practice. We systematically reviewed the evidence on the clinical and cost-effectiveness of shock-absorbing flooring use for fall-related injury prevention in care settings. We searched six databases, clinical trial registries, conference proceedings, theses/dissertations, websites, reference lists, conducted forward citation searches, and liaised with experts in the field. We conducted study selection, data collection, and critical appraisal independently in duplicate. We evaluated the influence of shock-absorbing flooring on fall-related injuries, falls, and staff work-related injuries. We adopted a mixed methods approach considering evidence from randomised, non-randomised, economic, qualitative, and implementation studies. We assessed and reported the quality of outcomes using the GRADE approach and Summary of Findings Tables. This review, conducted over the course of 2019, summarises the certainty of the evidence on whether and which shock-absorbing floors influence injuries from falls, the chance of someone falling over, and work-related injuries in staff (e.g. from manoeuvring equipment across softer floors). Our findings are applicable to health and social care professionals, buildings and facilities managers, carers, older adults, architects, and designers. Funded by National Institute for Health Research, Health Technology Assessment (ref 17/148/11); registered in PROSPERO (CRD42019118834).

